# The Persistent Challenge of Pneumocystis Growth Outside the Mammalian Lung: Past and Future Approaches

**DOI:** 10.3389/fmicb.2021.681474

**Published:** 2021-05-20

**Authors:** Melanie T. Cushion, Nikeya Tisdale-Macioce, Steven G. Sayson, Aleksey Porollo

**Affiliations:** ^1^Department of Internal Medicine, University of Cincinnati College of Medicine, Cincinnati, OH, United States; ^2^Medical Research Service, Cincinnati Veterans Affairs Medical Center, Cincinnati, OH, United States; ^3^Department of Pediatrics, University of Cincinnati College of Medicine, Cincinnati, OH, United States; ^4^Center for Autoimmune Genomics and Etiology, Division of Biomedical Informatics, Cincinnati Children’s Hospital Medical Center, Cincinnati, OH, United States

**Keywords:** Pneumocystis species, fungal pathogens, *in vitro* growth, Pneumocystis pneumonia, fastidious

## Abstract

The pathogenic fungi in the genus, Pneumocystis, have eluded attempts to continuously grow them in an *ex vivo* cultivation system. New data from transcriptomic and genomic sequencing studies have identified a myriad of absent metabolic pathways, helping to define their host obligate nature. These nutrients, factors, and co-factors are acquired from their mammalian host and provide clues to further supplementation of existing media formulations. Likewise, a new appreciation of the pivotal role for the sexual cycle in the survival and dissemination of the infection suggests that Pneumocystis species are obligated to undergo mating and sexual reproduction in their life cycle with a questionable role for an asexual cycle. The lack of ascus formation in any previous cultivation attempts may explain the failure to identify a sustainable system. Many characteristics of these ascomycetes suggest a biotrophic existence within the lungs of the mammalian hosts. In the present review, previous attempts at growing these fungi *ex vivo* are summarized. The significance of their life cycle is considered, and a list of potential supplements based on the genomic and transcriptomic studies is presented. State of the art technologies such as metabolomics, organoids, lung-on-a chip, and air lift cultures are discussed as potential growth systems.

## Introduction

Fungi in the genus, Pneumocystis, are host-obligate pathogens that can cause lethal pneumonia in mammals with impaired immune function, including humans. During the previous decades, HIV+ patients have made up the largest proportion of hospitalized cases of Pneumocystis pneumonia within the United States. However, recent studies have shown that malignancies now are the most prevalent host factor for hospitalizations with *Pneumocystis jirovecii* pneumonia accounting for 46% of cases in the United States compared to 17.8% with HIV+ as the underlying illness (Kanj et al., 2021). Moreover, a recent assessment of pneumonias in children 1–59 months in African and Asian countries revealed that *P. jirovecii* was a significant cause of infection, especially in children younger than 1 year of age (The Pneumonia Etiology Research for Child Health (Perch) study group, 2019). The increase in susceptible populations and the dearth of treatment options, signal a desperate need for new therapies. The search for new treatments for this fungal infection, as well as other aspects of investigation, have been hindered by the lack of a continuous *in vitro* cultivation system.

Such an *ex vivo* cultivation system remains elusive since the identification of these fastidious organisms over a century ago. As a result, the scientific community lacks a genetic system to explore gene function by knock- out/in technology; a drug screening assay that can discern pneumocysticidal vs static outcomes and one that can be used to screen the drug-induced phenotype of the infecting Pneumocystis species; as well as epidemiological studies that can identify drug resistant genotypes and track them throughout populations.

The origin of the Pneumocystis species was fraught with problems. It was first identified in 1909 as part of the life cycle of the parasite, *Trypanosoma cruzi*, in animals co-infected with the trypanosomes and Pneumocystis (Redhead et al., 2006). The pursuant attempts at taxonomic classification further exacerbated its valid identity by applying the International Code of Zoological Nomenclature rules for naming these microbes which resulted in acceptance of “Pneumocystis” as a zoonosis and thus one species, *Pneumocystis carinii*, could infect several mammalian species including humans and rats. The identity of Pneumocystis as fungal or protozoan was also controversial. Its true nature as a fungal pathogen was not fully resolved until well into the 21st century. In 2006, invalid names were eliminated, and the different species of Pneumocystis were validated with typification and named according to the Botanical Code of Nomenclature rules used for fungi (Redhead et al., 2006).

Gene and genome sequencing provided certain confirmation that “Pneumocystis” was a genus comprised of many species and each species was usually associated with a single mammalian host species. Efforts to correctly name the species led to the valid names and descriptions of 5 formally described species to date: *Pneumocystis jirovecii* which infects humans (*Homo sapiens*) (Redhead et al., 2006); *P. murina* which infects mice (*Mus musculus*) (Keely et al., 2004); *P. carinii* (Redhead et al., 2006) and *P. wakefieldiae*, that infect rats (*Rattus norvegicus*) (Cushion et al., 2004); and *P. oryctolagi* which infects rabbits (*Oryctolagus cuniculus*) (Dei-Cas et al., 2006).

Such efforts are not purely academic exercises, as understanding of a fungal or protozoan identity could suggest different approaches for cultivation outside the mammalian lung. Indeed, investigators applied techniques associated with the culture of either of these microbes as well as other tissue culture approaches, but none led to the “Holy Grail” of continuous passage and growth. This failure was not surprising considering the general lack of systematic assessments in the various trials and more importantly, the lack of understanding of the reduced metabolic capabilities of these host-obligate fungi.

Although little progress has been made in this area, strides in understanding the role of the life cycle and new technology provide avenues for further progress towards this goal. In this review, the previous attempts to cultivate these fungi will be summarized; the life cycle and implications on growth outside the lung will be discussed; the lack of metabolic capacity as revealed by genome sequencing will be examined; and novel, potential *in vitro* approaches will be presented.

## Previous Cultivation Attempts

Review of the published attempts to culture rodent-derived and human-derived Pneumocystis on cell monolayers and in cell -free media clearly reveal the lack of continuous passage and the abbreviated growth in primary (host-derived organisms that were not passaged) culture (Tables 1, 2). The assessment of “growth” varied widely, including microscopic enumeration with various tinctorial staining methods, ATP content, total DNA quantification and quantitative PCR methods to targeted genes, but *in toto*, there has been no system that has emerged as a reproducible method that has stood the test of time using any quantification technique. The failure of various laboratories when trying to replicate published methods has not been well documented in the literature as negative studies are not given priority in journals. Two studies garnered high interest; results reported by Merali et al. (1999) using Transwell inserts and Schildgen et al. (2014) using a novel cell line and an air-liquid interface system with CuFi-8 cells. Both reports were discounted when other laboratories conducted serious attempts at replication and were unsuccessful (Liu et al., 2018). Such publications are quite valuable to the community, avoiding lost time and expensive reagents. The most practical test would be the wide-spread adoption of a successful technique by many laboratories, which has clearly not occurred post-publication of any report.

**TABLE 1:** *In vitro* culture of Pneumocystis species with primary and cell lines.

**References/Year of publication**	**Source of inoculum**	**Cell type/lines supporting “growth”^*a*^ Medium/temperature**	**Quantification method**	**Maximal reported growth in one cycle/time of peak growth**	**Other cell lines reported as non-supportive of growth**
Pifer et al. (1977) 1977	*P. carinii* from immunosuppressed Sprague-Dawley rats lung wash *P. jirovecii*	Chicken embryonic epithelial lung cells (CEL); M99 with 10% FBS or M99 with 2% FBS, 35°C with 5% CO_2_	Enumeration of asci, toluidine blue O stain (TBO)	3 to 10-fold for *P. carinii;* 24-48 h. 10-fold* for *P. jirovecii;* 24-48 h.	AV3, WI-38, L cells, rat lung, secondary chicken embryo fibroblasts, owl monkey kidney, baby hamster kidney, Chang liver cells
Pifer et al. (1978) 1978	*P. carinii* from immunosuppressed Sprague-Dawley rat lungs shaken in PBS	Vero cell line; MEM with 2% FBS, 35^*o*^C with 5% CO_2_	Enumeration of asci, TBO	11-fold; 72 hours	ND
Latorre et al. (1977) 1977	*P. carinii* from immunosuppressed Sprague-Dawley rat lung homogenate	Vero, Chang liver, MRC-5 cell lines; Eagle’s Essential medium with 10% fetal calf serum; 37°C with no CO_2_	Visual estimation of the density of floating clusters of organisms	ND	LLC-MK-2, FL, McCoy
Bartlett et al. (1979) 1979	*P. carinii* from immunosuppressed Sprague-Dawley rat lung homogenate *P. jirovecii*	WI-38 and MRC-5 cell lines; Eagle’s essential medium with 10% fetal calf serum; 35°C with no CO_2_	Enumeration of Giemsa-stained “trophozoites”	13-fold; 4 -8 days for *P. carinii* on both cell lines No growth with *P. jirovecii*	ND
Cushion et al. (Cushion and Walzer, 1984; Cushion et al., 1985) 1984/1985	*P. carinii* from immunosuppressed Sprague-Dawley rat lung homogenate *P. jirovecii* from bronchoalveolar lavage and tissue (BALF)	A549 cell line Dulbecco’s modified Eagle medium (DMEM) with 10% inactivated FBS at 37°C with 5% CO_2_ WI-38 VA 13 Subline 2RA HMEM, 25 mM HEPES, 10% FBS, 2X MEM vitamin solution, 1X non-essential amino acid solution 37°C with 5% CO_2_	Rapid Giemsa (Diff-Quik^TM^) for enumeration of nuclei/ml; cresyl echt violet (CEV) for asci	(A549) 10-fold; Day 7; (WI-38 VA13 Subline 2RA) 20-fold. (A549) 10-fold; Day 14 with 1 of 10 isolates. ND for WI-38 VA13 subline 2RA	WI-38, L2, 4/4 RM4, RFL-6, Hep-2
Smith and Bartlett (1984) 1984	*P. carinii* from immunosuppressed Sprague-Dawley rat lung homogenate	Walker (LLC-WRC 256) MDBK HeLa LU-1 MCF-7	Enumeration of Giemsa-stained “trophozoites”	NG^∗∗∗^	ND
Armstrong and Richards 1989	*P. carinii* from immunosuppressed Sprague-Dawley rats lung wash	Mv 1 Lu cell line HEPES buffered Eagle’s MEM with Earl’s salts with 1X non-essential amino acids, 1% sodium pyruvate, 1% L -glutamine, and 10% heat-inactivated FCS; 37°C with 5% CO_2_	Giemsa stain for enumeration of all stages	2-6-fold Days 4 to 8	
Cushion and Walzer (Cushion, 1989) 1989	*P. carinii* from immunosuppressed Sprague-Dawley rat lung homogenate	Fetal organotypic culture Lung explant culture 37°C, 5% CO_2_	Rapid Giemsa (Diff-Quik^TM^) for enumeration of nuclei/ml; cresyl echt violet (CEV) for asci	NG	
Bartlett et al. (1992) 1992	*P. carinii* from immunosuppressed Sprague-Dawley rat lung homogenate	Human embryonic lung cells; MEM without serum; 35°C in 5% CO_2_	Giemsa stain for enumeration of “trophozoites:	3-fold; 7 days	
Aliouat et al. (1999) 1999	*P. carinii* from immunosuppressed rat lung homogenates	L2 cells; DMEM 10% heat-inactivated fetal calf serum; 37°C with 5% CO_2_	TBO for asci, RAL555 for trophozoites and “filled” asci	2-4-fold	
Cirioni et al. (1997, 2000) 1997/2000	*P. jirovecii* from BALF	A549 cell line; DMEM with and without a serum supplement of 10% FCS; Fe(NO)_3_; L-glutamine, HEPES; 37°C with 5% CO_2_	Giemsa for enumeration of “trophozoites: and methenamine silver for asci	3-fold; 72 h.	
Atzori et al. (1998) 1998	*P. carinii* from immunosuppressed rat lung homogenate	L2; Dulbecco’s modified Eagle’s medium, 5% CO_2_, 37°C	RAL 555 (Rapid Giemsa-like stain) for all stages except asci; toluidine blue O for asci	2-4-fold	
Ni and Chen (2001) 2001	*P. carinii* from immunosuppressed rat lung homogenates	HepG-2 cell line;	Diff-Quik^TM^ for “trophozoites” Gomorri’s methenamine silver stain for asci	5 days	
Schildgen et al. 2014**	*P. jirovecii* from BALF	CuFi-8 cell line; Bronchial epithelial cell basal medium (BEBM) grown on human placental collagen in flasks then transferred to transwells with Ham’s F12K medium; 37°C	qPCR targeting the mitochondrial large subunit rRNA (mtLSU) and Major surface glycoprotein (MSG)	Up to 3 log_10_ units for both targets; 5 days	

*Not apparent from the presented data. **According to the publication by Kovacs et al., this system could not be duplicated (Liu et al., 2018). ***No Growth.

**TABLE 2:** *In vitro* culture of Pneumocystis species in cell free media.

**References/Year of publication**	**Source of inoculum**	**Medium/temperature**	**Quantification method**	**Maximal reported growth in one cycle/time of peak growth**
Pifer et al. (1977) 1977	*P. carinii* from immunosuppressed Sprague-Dawley rats lung wash	Balamuth’s egg extract medium Blair’s medium Chang’s medium Diamond’s medium Newton’s medium NNN medium Pan’s medium Schaedler’s broth Tobie’s medium All were at 35°C	Enumeration of asci, toluidine blue O stain	NG*
Tegoshi and Yoshida 1989	*P. murina* from nude mice	L15 or DMEM 10% heat-inactivated FBS, 100 μM 2-mercaptoethanol, 50 μM bathocuprine sulphonate; cysteine was added daily (quantity not specified) 37°C, 5% CO_2_	Enumeration, Giemsa stain	4-10 fold; L-15 showed slightly better results
Cushion and Ebbets (1990) 1990	*P. carinii* from immunosuppressed Lewis rat lungs	DMEM Yeast extract-peptone-dextrose (YEPD) broth and agar Yeast extract-malt extract (YM) Brain Heart Infusion broth and agar Wort broth and agar Sabourad’s broth and agar Vogel and Johnson agar Physiological saline, phosphate buffered saline, Hanks balanced salt solution. Solid agars tested at 4.0 and 7.0 pH. 1% neopeptone (wt/vol) with 0.2% (wt/vol)	Diff-Quick stain for all life cycle stages	NG
			N-acetylglucosamine (NPG) at pH 4.0, 5%CO_2_, 37°C**	8-10-fold
Aliouat et al. (1999) 1999	*P. carinii* from immunosuppressed Wistar rat lungs	DMEM with 10% FCS, 5%CO_2_, 37°C	TBO, RAL555 for “trophozoites” and “filled” asci	2-fold
Merali et al. (1999) 1999***	*P. carinii* from immunosuppressed rat lungs	Minimal Essential Medium with Earle’s salts (MEME) with 20% horse serum, 500 μg/ml, S-adenosyl-methionine sulfate (twice per day), 80 μg/ml of p-aminobenzoic acid, putrescine, ferric pyrophosphate, L-cysteine, L-glutamine, and N-acetyl-glucosamine with penicillin and streptomycin; 31°C, normal atmosphere *P. carinii* were inoculated in 0.4 μM pore, collagen coated Transwells and suspended in the medium described above.	DNA stained with Hoechst dye 33258 and analyzed using an HPLC system.	130- to 1000-fold
Lasbury et al. (1999) 1999	*P. murina* and *P. carinii* from immunosuppressed lungs	Medium as above (Merali et al., 1999) in Biocoat cell culture inserts	Giemsa for “trophozoites”	12-fold for *P. murina*; 14-fold for *P. carinii*
Sobolewska and Dzbenski (2009) 2009	*P. carinii* from immunosuppressed Wistar rat lungs	MEME with 10% horse serum, 500 μM twice per day S-adenosyl-methionine sulfate, 80 μg/ml of p-aminobenzoic acid, putrescine, ferric pyrophosphate, L-cysteine, L-glutamine, and *N*-acetyl-glucosamine with penicillin and streptomycin; 31°C, normal atmosphere Inoculated in 0.4 μM pore, collagen coated Transwells and suspended in the medium described above	Giemsa and Diff-Quick for asci; PCR directed to the mtLSUrRNA	257-fold increase in asci; 286-fold with RT-PCR
Cushion et al. (2009)	*P. carinii* from immunosuppressed rat lungs *P. murina* from immunosuppressed mouse lungs Both from cryopreserved aliquots	RPMI 1640 medium, penicillin- streptomycin, amphotericin B, vancomycin, 20% calf serum, vitamins and non-essential amino acids as described (Cushion et al., 2006) PET track-etched membrane cell culture inserts, Transwell, and Millicell-CM hydrophilic (PTFE)# membranes	ATP content by bioluminescence; β-1,3-D-glucan levels	8-10-fold; peak at Day 10 for *P. carinii;* not reported for *P. murina.* 61–75% increase from Day 1 to Day 21 for both species

*No Growth. **A variety of supplements (carbohydrates, nitrogen sources, sterols, fatty acids, misc., gas mixtures, temperatures and pH were tested in NPG liquid medium without further improvement of growth (Cushion and Ebbets, 1990). ***Larsen et al. (2002) found no organism replication using a quantitative touchdown PCR assay. ^#^Best supported biofilm formation.

Mostly guided by requirements of other fungi or pathogens, culture attempts have relied on cell monolayers flooded by liquid media or cell-free media to which the Pneumocystis species (spp.) are directly inoculated. Neither environment adequately mimics the unique location in the lung where these fungi grow in the “hypophase” of the alveoli which is a thin continuous layer of about 200 nm that likely covers the entirety of the alveolar surface (Fehrenbach, 2001). The composition of the hypophase includes pulmonary surfactant which is regulated by pH and Ca^2+^ proximally and provides low surface tension. The Type II pneumocyte or Alveolar Epithelial Cell Type 2 (AEC2) secretes a myriad of other factors including epidermal growth factor, VEGF, adhesion molecules, lipids (especially dipalmitoyl phosphatidylcholines) entactin, laminin, fibronectin, and proteoglycans. The presence of this liquid lining layer means the alveolar epithelial cells (AEC1 and AEC2) are not directly exposed to air (Knudsen and Ochs, 2018). Most of these factors have been added to one *in vitro* system or another, without success.

## The Proposed Life Cycle

**FIGURE 1:**
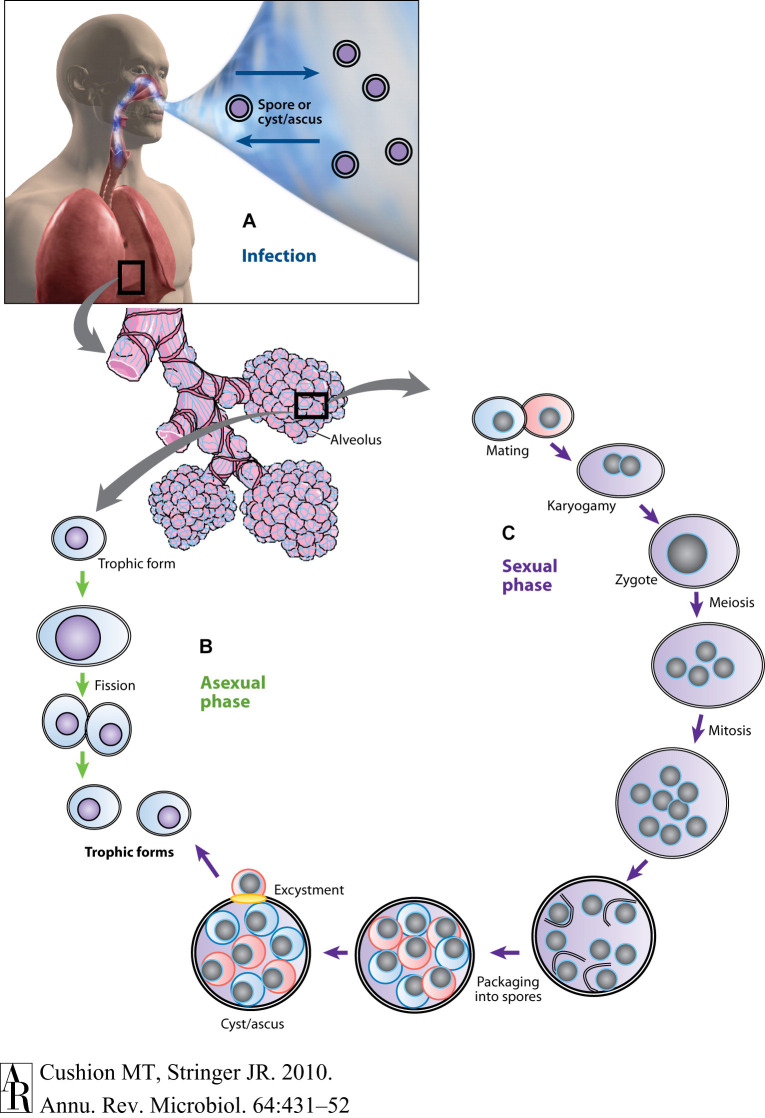
Putative life cycle of Pneumocystis. **(A)** Infection. Recent studies suggest that the cyst/ascus (containing eight spores) is the agent of infection (inward arrow). After inhalation, the spores ultimately take residence in the terminal portion of the respiratory tree, the alveoli (enlarged bundles of alveoli shown in the illustration). Neither the mechanism of migration to the alveoli nor the form in which the organism arrives in the alveoli (intact ascus or individual spores) is known. It is speculated that the spores are released by exhalation (outward arrow). **(B)** Asexual phase. Haploid trophic forms are thought to replicate asexually by binary fission, a process whereby a single trophic form duplicates its genetic material and creates two daughter forms of roughly equal sizes. **(C)** Sexual phase. Two presumptive mating types conjugate, undergo karyogamy, and produce a diploid zygote that progresses through meiosis and then an additional mitosis to produce eight nuclei. The nuclei are packaged into spores by invagination of the ascus cell membranes. After completion, excystment occurs via a protunicate release by unknown mechanisms, which may involve a pore or opening in the cyst wall (yellow oval). The released spores become the vegetative forms, the haploid trophic forms, that can then undergo asexual or sexual replication (Images of man, alveoli purchased from Superstock Photos, http://www.superstock.com). Annual Reviews Authors: There is no need to obtain permission from Annual Reviews for the use of your own work(s). Our copyright transfer agreement provides you with all the necessary permissions. Our copyright transfer agreement provides: “…The nonexclusive right to use, reproduce, distribute, perform, update, create derivatives, and make copies of the work (electronically or in print) in connection with the author’s teaching, conference presentations, lectures, and publications, provided proper attribution is given…”

A better understanding of the life cycle and the host obligate nature of these microscopic fungi are essential to the Pneumocystis ouroborous that may include an asexual cycle, a sexual phase, an immune- debilitated mammalian host and dissemination of the infection. First guided by images provided by light-, fluorescent-, and transmission electron microscopy, proposed life cycles included asexual replication via binary fission; asexual and sexual replication leading to the production of asci (once referred to as “cysts”); exit of the asci from the host; and release of spores (“daughter forms”) to initiate infection in a new host. A distillation of these hypotheses and proposed life cycle stages are shown below, Figure 1. Recent studies using molecular technology such as RNA-seq and high throughput sequencing have provided strong evidence for a sexual cycle in the lung with primary homothallism as the mode, but these and other reports have cast doubt whether an asexual cycle is necessary or even operational (Hauser and Cushion, 2018). That the ascus is the agent of transmission was shown by experiments in the mouse model of Pneumocystis infection (Cushion et al., 2010). Treatment with commercially available echinocandins does not eliminate Pneumocystis pneumonia, rather these drugs inhibit the ability of Pneumocystis to produce asci, owing to their inhibition of β-1,3-D-glucan biosynthesis. Our laboratory showed that these mice without asci, but with large organism populations that did not produce -β-1,3-D-glucan, were unable to transmit the infection. Inoculation of these same organisms into *P. murina*-naïve and immunosuppressed mice were able to reconstitute the infection with the re-emergence of asci.

## Biofilm Formation

**FIGURE 2:**
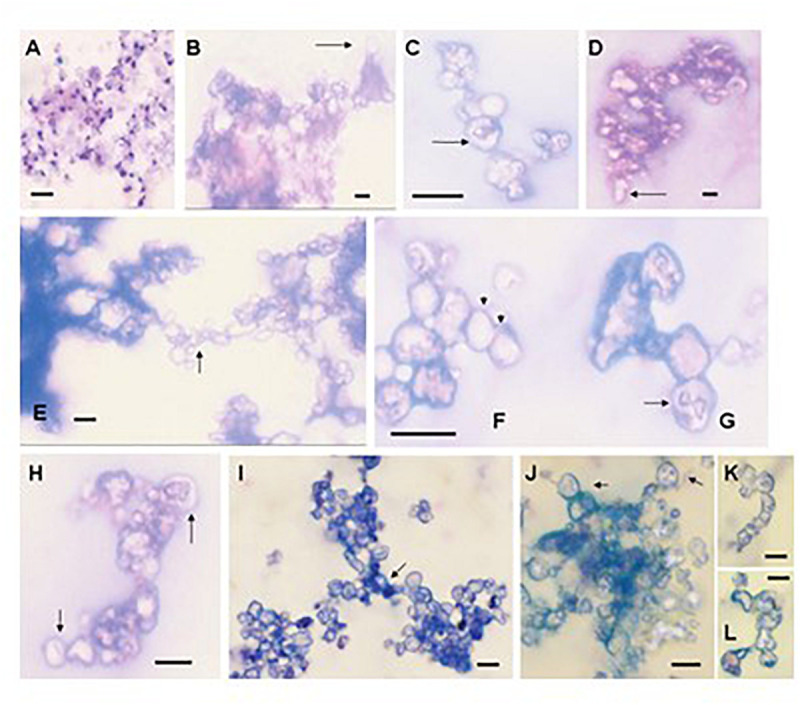
The morphology of Pneumocystis changes dramatically during biofilm formation. **(A)** *P. carinii* from the supernatant of a 3-day-old standard short-term culture stained with Hema3, illustrating the differences in morphology from the biofilm structures. **(B–H)** Images were taken from 16-day-old biofilms inoculated with *P. murina* (obtained as a fresh isolate). The images were obtained from films on inserts that were scraped with a pipette tip, aspirated, air dried, and stained with Hema3, a rapid Wright-Giemsa stain. Images were viewed under oil immersion. Bars, 10 m. **(B)** *P. murina* cluster showing a cyst-like structure with a stalk (arrow). **(C)** An ascus in a chain with intracystic forms. **(D)** Refractile cluster beginning to form extensions (arrow). **(E)** Two large clusters forming a linkage or bridge structure (arrow). **(F)** A series of cyst-like structures forming a chain (arrows). **(G)** Cyst containing a structure which appears to be trophic forms undergoing binary fission or conjugation (arrow). **(H)** Ascus-like forms containing intracellular spore-like morphologies (arrows). **(I–L)** Images were taken from *P. carinii* biofilms at day 9 of the first passage. **(I)** Cluster mass seemingly comprised of three clusters that have joined in the center (arrow). **(J)** Ascus-like structures in a cluster with obvious intracellular contents resembling spores (arrows). **(K,L)** Chain-like forms. Bars, 10 μm (Cushion et al., 2009).

Formation of biofilms is a strategy used by pathogenic microbes as well as microbes found throughout the environment. Organized microbial communities of fungi and bacteria attach to biotic or abiotic matrices to exchange plasmids, reduce susceptibility to antimicrobial agents and/or host immune responses or to protect members from other environmental stresses. These structures are also used to seed environments by dispersion from the community. Many pathogenic fungi utilize biofilms to survive within the host environment including Candida spp. (Ramage et al., 2001a,b; Mowat et al., 2007), *Cryptococcus neoformans* (Martinez and Casadevall, 2007), and *Aspergillus fumigatus* (Mowat et al., 2007). The structure of the Pneumocystis cells with the alveoli of the lung follows the characteristics of a biofilm as they are closely enmeshed, contain exopolymeric components, and spread throughout the lung (Cushion et al., 2009). We showed that *P. carinii* and *P. murina* could produce macroscopically visible and reproducible biofilms on inserts composed of hydrophilized PTFE (Biopore-CM from Millipore) and Millicell-HA cellulose (Cushion et al., 2009). These fungi rapidly formed tightly adherent biofilms that were able to maintain ATP levels over 2-3 weeks. Notably, the organisms completely changed morphology in biofilms. Figure 2A shows an organism cluster from an RPMI-1640-based cell-free culture stained with a rapid variant of the Wright-Giemsa stain. Note the changes in structure as the biofilm matures (Figures 2B–L). Although all samples were stained with Wright-Giemsa, many structures excluded the dyes and were refractile under light microscopy.

The biofilm cultures were then used to assess the effects of drugs on their ATP content. ATP measurement is used as a surrogate read out for viability (Cushion and Collins, 2011). These studies revealed several differences between the standard cell free suspension cultures and the biofilms. *P. carinii* organisms were more resistant to the echinocandins in mature biofilms than those in the RPMI 1640-based cell free suspension assay. Newly forming (nascent) biofilms were more susceptible than established biofilms and the populations in the nonadherent phase of the biofilms were generally more susceptible to echinocandin activity than the adherent populations. Notably, higher serum concentrations (10–20%) abrogated the efficacy of the echinocandins, especially anidulafungin, in suspension or biofilm assay systems. Exposure to anidulafungin consistently and significantly reduced the ATP levels than did caspofungin or micafungin in either *in vitro* assay system. Though promising, the biofilm cultures failed to propagate outside the primary culture.

## What Elements Are Missing From All These Cultivation Efforts?

The systems summarized above and other unreported failures to identify an environment where these fungi can thrive, “begs the question-” what was lacking in these myriad of culture methods? With some exceptions, there has not been many systematic evaluations of media and supplements for Pneumocystis spp. growth. The standard approach of adding supplements in limited concentrations based on various rationales has not proven to be fruitful. Our laboratory embarked on a yearlong study supported by the United States National Institutes of Health, to systematically evaluate nutrients, trace metals, lipids, co-factors, and other compounds. We used an RPMI 1640-based cell free system with a 10–20% serum supplement, which seems to be indispensable for viability in our hands. In some cases, the rationale for supplements was based on a comparative genomics study that revealed the lack of ability to synthesize vital compounds, e.g., *myo*-inositol Porollo et al., 2014; Cushion et al., 2016), while others were more exploratory in nature, e.g., trace metals. Our experiences revealed a lack of batch-to-batch consistency from the cryopreserved Pneumocystis spp. we used. However, the addition of *myo-*inositol most consistently improved the ATP content vs the un-supplemented cultures.

Another apparent constancy from most of the studies was the peak of replication, growth, and viability early in primary culture after a few days in culture, with declining values thereafter. Such a clear signal suggests the following: (1) the small increases could be explained by a “coasting” of the Pneumocystis spp. life cycle stages, where some stages completed a replication or there is a spore release from asci using intracellular nutrients previously gleaned from the host environment; (2) the artificial media did not replenish critical nutrients or the concentrations were insufficient or at levels to be inhibitory to further growth; (3) there was no asci production, leading to a cessation of the life cycle; (4) the total environment cannot sustain replication, lacking a sufficient carbon dioxide level, support phase (liquid, gel, solid, e.g.), or substrate; (5) stimuli for asci production/sexual reproduction were absent. This last point is likely critical for a sustainable culture system, as we have shown recently that the sexual cycle is required for replication in rodent models of Pneumocystis infection (Cushion et al., 2010; Miesel et al., 2021).

Much of the life cycle of these fungi occurs in the mammalian lung and specifically in the alveoli. Within the alveoli, Pneumocystis preferentially and specifically attaches to the AEC1 cells which raises the question, why? Perhaps there are certain receptors on its surface that forms the tight interdigitation with these fungi. If so, what might be the purpose? Two thoughts come to mind that are not mutually exclusive. One is that there is an intimate exchange of nutrients that Pneumocystis requires. The second suggests a more cunning reason. By binding tightly to the very cell necessary for gas exchange, the fungal parasite directs a change in its immediate environment by altering the gas mixture, favoring a more hypoxic environment. There is some evidence that supports this hypothesis. The Pneumocystis genomes lack homologs to carbonic anhydrase genes (Ma et al., 2016). Carbonic anhydrases catalyze the interconversion between carbon dioxide and water and the dissociation of carbonic acid, bicarbonate, and hydrogen ions. These enzymes maintain acid -base balance and helps to transport carbon dioxide. In the lungs, carbon dioxide is being released, so its concentration is lower than in tissue. The yeast genome contains a carbonic anhydrase encoded by NCE103 (Martin et al., 2017). The nce103 null mutant exhibits impaired growth under aerobic conditions, as do bacteria lacking these enzymes However, growth under these conditions can be restored if augmented with high levels of carbon dioxide, which apparently satisfies the need for bicarbonate formation supplied by the carbonic anhydrases. Might these obligate fungal pathogens be facilitating their survival by increasing the carbon dioxide in their immediate environment? There is some evidence that this might occur. Our laboratory explored the effects of 3 different gas mixtures on the ATP content of *P. carinii* in an RPMI 1640-based medium (Joffrion et al., 2006). A significant increase in ATP content was observed for fungi grown under microaerophilic conditions (10–15% O2; 7–15% CO2) when compared to standard conditions of 5% CO2. Anaerobic conditions resulted in sharp decreases of ATP by 24 h, suggesting that oxygen is required. Interestingly, these different atmospheres resulted in distinct responses to trimethoprim-sulfamethoxazole. Whereas the ATP levels of *P. carinii* in standard medium with 5% CO2 had decreased levels of ATP by 75% with treatment, those treated under microaerophilic conditions fell by only 50% vs the untreated controls. While the increase in carbon dioxide levels did not permit continuous culture, such a condition may play a key factor in future experiments with a more supportive culture structure. It should also be noted that the pulmonary epithelial pneumocytes do not have detectable carbonic anhydrases on their apical surface, accessible to the fluid lining, but activity is very abundant on the pulmonary endothelial cell surface facing the plasma (Effros, 2008). Thus, in such a bicarbonate starved environment, perhaps these fungi can find an alternative mode of acquisition.

The recent publications of the genomes of *P. carinii, P. murina*, and *P. jirovecii* identified metabolic cycles and pathways that are lacking in these fungi and are likely to lead to more rational supplementation studies (Cisse et al., 2014; Ma et al., 2016). Concomitant with such insights is the requirement for a balance of the supplement concentrations. Advances in metabolomics technology can suggest appropriate levels based on metabolic flux analyses while newer approaches to cell-based culture, e.g., air lift cultures, alveolar organoids, offer alternatives not available until recently. Lastly, a better understanding of the life cycle of these fungi and their apparent reliance on sexual reproduction strongly suggest that factors which stimulate this mode of replication needs to be considered in future *in vitro* culture systems. These considerations are discussed below.

## Biotrophy

Several publications have revealed the host-obligate nature of Pneumocystis spp. (Cushion et al., 2007; Cisse et al., 2014; Hauser, 2014; Porollo et al., 2014; Ma et al., 2016). An early analysis of Expressed Sequence Tags (ESTs) from infected rat lungs demonstrated the absence of critical genes in such pathways as the pyruvate bypass and glyoxylate cycle, but the presence of genes necessary for carbohydrate metabolism, and suggested that Pneumocystis spp. may be obligate biotrophs (Cushion et al., 2007).

Biotrophy has been observed in fungi that invade plants, derive their energy from the host cells, but do not kill them. Obligate biotrophs complete their entire life cycle within the plant host, including the sexual cycle, and are incapable of growth outside the host (Lorrain et al., 2019). Pneumocystis spp. only grow within the lungs of mammals and complete their life cycle therein; are unable to grow outside the lungs (at present); and do not invade host cells. Pneumocystis do not produce hyphae in the lung and maintain an extracellular existence. Thus, Pneumocystis spp. fulfills these elements for biotrophic existence, including perhaps the lack of pathogenic effects. Though Pneumocystis spp. cause disease in immunocompromised hosts, it is currently held that mammals with intact immune systems are often transiently infected and may even enter a commensal lifestyle with the fungi without associated illness. It is only when the host tips the balance and losses its ability to control the infection that the organisms grow unchecked causing the disease state. It is also well known that Pneumocystis spp. grow slowly, even in severely immunocompromised hosts, suggesting they have adapted well to their hosts and are averse to killing them.

**FIGURE 3:**
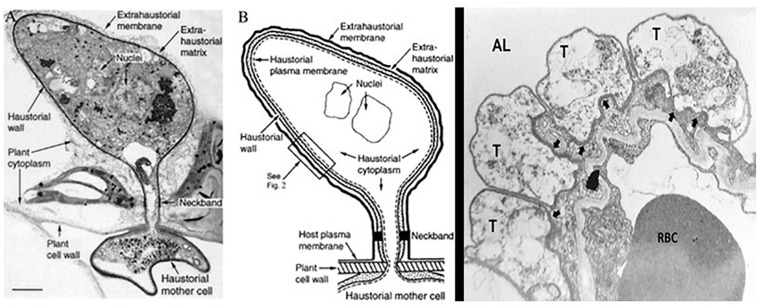
Haustorial complex, a specialized feeding organ of biotrophic fungal parasites of plants. To move from host cell to fungus, nutrients must traverse the extrahaustorial membrane, the extrahaustorial matrix, the haustorial wall, and the haustorial plasma membrane. A neckband seals the extrahaustorial matrix from the plant cell wall region so that the matrix becomes a unique, isolated, apoplast-like compartment. The haustorium connects to intercellular fungal hyphae by way of a haustorial mother cell. A proton symport system in the haustorial plasma membrane drives sugar transport from plant to parasite. **(A)** Transmission electron micrograph of a flax rust haustorium [Reproduced with permission from ref. 2 (Copyright 1972, NRC Research Press)] (Bar, 1 μm). **(B)** Drawing showing key features of the fungal haustorium. **(C)** Electron micrograph of Pneumocystis trophic forms in tight apposition with the AEC1 in the lung alveoli. Trophic forms (T); Alveolar Lumen (AL); Red Blood Cell (RBC); arrows indicate the tight apposition of the trophic form double membrane with the AEC1 membranes. From the collection of MTC.

Another intriguing attribute of some obligate biotrophy in fungi such as plant pathogenic rust fungi is the network of intercellular hyphae called haustoria (Struck, 2015). Haustoria are structures considered to facilitate the acquisition of nutrients from the host cell. Haustoria form after the fungi penetrates the cell wall of the plant cell but do not wound the plant plasma membrane (Figure 3A). The haustoria grow in the living plant cells and are in intimate contact with the plant cell cytoplasm (Figure 3B). The cytoplasm of the host and fungus are separated by the host plasma membrane, the fungal plasma membrane and a matrix called the “extrahaustorial matrix” (Figures 3A,B). The species within the genus Pneumocystis are not known to produce hyphae, like many other fungi. Rather, they are relegated to the external mammalian lung environment. It is not difficult to imagine that the intimate interaction of the Pneumocystis trophic form with its alveolar host cell (Figure 1C) may be similar to that of the obligate biotrophic fungi with a structure that could conceivably facilitate nutrient acquisition. Early studies reported activation of the plasmalemmal vesicular system in the alveolar cells associated with trophic forms near the site of attachment to the AEC1 (Settnes and Nielsen, 1991), suggesting an approximation of a feeding complex. The trophic forms have long been considered the vegetative form of these fungi and this complex could be a novel manner of acquiring necessary nutrients lost during evolution of their parasitism.

## Loss of Genes in Metabolic Pathways Suggest Extensive Supplements Are Necessary

Comparative genomic analyses do not suffer from problems associated with the analysis of the transcriptome, such as transient expression of genes involved in metabolic pathways. Using genomic analyses, Cisse et al. documented a dearth of enzymes necessary for amino acid synthesis in *P. carinii* (Cisse et al., 2014), with only 2 enzymes representing potential aspartic acid and glutamic acid synthesis as compared to *Schizosaccharomyces pombe*’s 54 enzymes involved in amino acid biosynthesis. While our study also did not detect any gene homologs involved in isoleucine, leucine, lysine or valine biosynthesis, homologs for phenylalanine, tyrosine, and tryptophan biosynthesis were identified in all 3 species (Porollo et al., 2014). In addition, homologs for metabolism of glycine, serine, threonine, alanine, aspartate, glutamate, arginine, proline, histidine, tyrosine, phenylalanine, and tryptophan were detected as well as for valine, leucine, isoleucine, and lysine degradation. Counter-intuitive to the need for host amino acids, Ma et al. reported a dramatic reduction in amino acid permeases in the 3 sequenced genomes when compared to free-living and pathogenic fungi: 1 in each Pneumocystis genome vs 10 to 32 in the others (Ma et al., 2016). This finding could mean that the single permease in Pneumocystis is promiscuous or there are other means to import their amino acid requirements. In fact, Ma et al. notes that the number of transcription factors and transporters in Pneumocystis are among the lowest in fungi (Ma et al., 2016).

In a study which compared the genomes of *P. jirovecii, P. murina* and *P. carinii* with *S*. *pombe*, we noted the absence of inositol-1-phosphate synthase (INO1) and inositol monophosphatase (INM1) from all the Pneumocystis genomes as well as from *S. pombe* (which had previously been reported) (Porollo et al., 2014). These genes encode the enzymes necessary for *myo-*inositol synthesis, a compound necessary for life in eukaryotes. This notable absence led us to the identification homologs to the *S. pombe* inositol transporter genes (ITR1 and ITR2) and the characterization of a highly specific *myo-*inositol transporter in the genomes of all 3 species (ITR1) with a second homolog (ITR2) in the 2 rodent species (Porollo et al., 2014; Cushion et al., 2016). Notably, there were increased genes in inositol phosphate metabolism, starch and sucrose metabolism, and amino sugar and nucleotide sugar metabolism when compared to the genome of the free-living fission yeast. Also of interest was the 5 additional genes encoding enzymes in the tryptophan metabolism pathway in *P. carinii* and *P. murina*, which were absent in *P. jirovecii* (Porollo et al., 2014). To evaluate *myo-*inositol auxotrophy, supplementation studies of *myo-*inositol to both rodent species in a cell free in vitro system resulted in a notable increase in ATP and a longer period of viability (Porollo et al., 2014). Table 3 is a compilation of predicted nutritional supplements or conditions from published genomics studies.

**TABLE 3:** Predicted nutritional supplements indicated by absent genes and pathways.

**References**	**Pneumocystis species**	
**Amino Acids**	
Hauser et al. (2010)	*P. carinii*	Alanine
		Asparagine
		Arginine
		Cysteine
		Glutamine
		Glycine
		Histidine
		Isoleucine
		Leucine
		Lysine
		Methionine
		Phenylalanine
		Proline
		Serine
		Threonine
		Tryptophan
		Tyrosine
		Valine
**Vitamins**		
(Hauser, 2014)	*P. carinii, P. murina, P. jirovecii*	Thiamine (B1)
Cushion MT	*P. carinii, P. murina*	Biotin (H, B_7_, B_8_)
**Other**		
Porollo et al. (2014)	*P. carinii, P. murina, P. jirovecii*	*myo-*inositol (carbocyclic sugar)
(Hauser, 2014)	*P. carinii, P. murina, P. jirovecii*	Nitrogen and sulfur assimilation
		Purine degradation
Joffrion et al. (2006)	*P. carinii, P. murina, P. jirovecii*	Increased carbon dioxide atmosphere
Ma et al. (2016)	*P. carinii, P. murina, P. jirovecii*	Absence of polyamine biosynthesis; evidence of a polyamine transporter suggests host acquisition
		Glucose
		Choline
		Ether lipids, complex sphingolipids, phosphatidylinositol, phosphatidylcholine, fatty acids
		Glycerol
		Pantothenate
		Ubiquinone/coenzyme Q
		Siderophores
	*P. jirovecii* only	Nicotinamide adenine dinucleotide
Ma et al. (2016)	*P. carinii, P. murina* (but not *P. jirovecii*)	Cholesterol
Furlong et al. (1997)	*P. carinii*	Fatty acids, cholesterol
Worsham et al. (2003)	*P. carinii*	Cholesterol

## Sexual Reproduction Holds the Key for *in vitro* Replication

Until recently it was assumed by most of the scientific community that like other fungi, Pneumocystis species could replicate asexually and sexually (Vossen et al., 1977; Vossen et al., 1978; Cushion, 2010; Skalski et al., 2015). Attempts to propagate these fungi *in vitro* typically led to apparent increases in the trophic form numbers and it was accepted that asexual replication was the preferred mode in these less-than-optimal culture systems. Other microbes like *Giardia duodenalis*, provided precedent in this thinking as only the trophozoites of these protozoans can be cultured *in vitro* (Perrucci et al., 2019) and those in the field settled with this shortcoming. With more current studies illustrating the reliance on the sexual cycle and asci formation as required for proliferation (Cushion et al., 2018), the absence of sexual replication in any *in vitro* system may be a key reason for lack of continuous growth. Notably, the use of a long-acting echinocandin, rezafungin, as well as anidulafungin and caspofungin when given in a prophylactic model could prevent the infection (Miesel et al., 2021). Thus, we surmise that not only are these fungi dependent upon the host for the metabolic requirements they can no longer synthesize, but they must also undergo sexual replication to survive.

## Insights for Future Success

Thoughtful re-consideration of the previous attempts to propagate Pneumocystis species outside the mammalian lung environment indicates a clear role for a host cell component. Alveolar cells may offer an initial substrate for the newly released trophic forms from the asci or they may contribute directly to the nutrient pool via an intimate feeding structure as suggested above. Alternatively, secreted molecules from the alveolar cell encapsulated in extracellular vesicles or exported through simple exocytosis may also provide another source of required compounds. In our laboratory, we are exploring both two-dimensional and three-dimensional cellular based systems as potential growth systems. We provide some preliminary results and discussions below.

## Three-Dimensional Alveolar Organoids

Methodology for the routine culture of enteric organoids is now quite advanced, but alveolar organoids remain a challenge (Li et al., 2020). Our laboratory explored a mouse lung cell organoid approach for anticipated inoculation of *P. murina*, the species that infects the mouse. This reasoning was based on evidence of species specificity of Pneumocystis for its mammalian hosts, e.g., *P. carinii* only infects rats, *P. murina* only infects mice. Results were promising and will be published in a separate report.

## Air-Liquid Interface Cell Cultures

Air liquid interface (ALI) cultures are used for respiratory research. Both primary cells from donors and immortalized cell lines have been used. The ALI system is defined by contact of the basal surface of the cells with liquid culture medium while the apical surface is exposed to air. Such a juxtaposition permits differentiation, as in the case of human bronchial epithelial cells transitioning to a pseudostratified mucociliary phenotype. To initiate the cultures, cells are typically placed onto a permeable membrane of a cell culture insert with medium within the insert and in the cell well below. Once confluent, the medium is only provided to the basal chamber, causing the “air lift” of the cells in the insert. Indeed, Schildgen et al. used such an ALI system with human airway cells, CuFi-8, which were derived from the bronchus of a patient with cystic fibrosis (Schildgen et al., 2014). As in previous attempts, *P. jirovecii* could not be continuously cultured in this system and in this case, it may have been due to the type of cell used for the ALI, bronchus derived cells rather than alveolar epithelial cell.

Our laboratory used this approach with the A549 cell line (ATCC CCL 185) which is described as an adenocarcinomic human alveolar basal epithelial cell derived from cancerous lung tissue of a 58-year-old Caucasian male (Giard et al., 1973). After air lift, the cultures were inoculated with *P. murina*, since the species infecting humans, *P. jirovecii* is not widely available. There was not a high expectation for growth, but this was rather an observational exercise. Somewhat surprisingly, the A549 cultures produced AEC1-like cells that stained with podoplanin, a marker for this cell type, and not with Surfactant Protein B, a marker for AEC2 cells. Staining with a marker specific for *P. murina*, the Major Surface Glycoprotein (Msg) superfamily of surface antigens, we could show that the fungi were attached to the apical surface and to the AEC1 cells. An optimal ALI would use host cells from the natural host wherein the Pneumocystis species reside. This approach demands more investigation as a potential growth system.

### Alveolar Lung Chips

Significant advancements in lung-chip models also offer promise. Such systems are commercially available and offer support platforms for epithelial and vascular channels, cell-cell interactions, immune cells, extracellular matrix and mechanical forces (Benam et al., 2016). Importantly for the potential growth of Pneumocystis spp., alveolar epithelial cells (AEC) can maintain AEC1 and AEC2 cell structure with expression of specific markers. Such systems can be used to evaluate drug response and inflammatory responses and may even be able to provide insights into factors responsible for species specificity. One drawback is that these systems are currently limited in their life span, which is about 2 weeks.

## Metabolomics

Metabolomics is a growing field that enables the unbiased detection and measurement of metabolites resulting from cell metabolism. Commonly, NMR spectroscopy is used to build profiles of the physiology of cells at the time of sampling. Application of this technology to identify and assess the utilization of host products by Pneumocystis in cell culture or even *in vivo* should provide additional detailed information related to cell cycle requirements of these obligate fungi. Our lab used H-NMR spectroscopy to assess the dynamics of uptake and secretion of metabolites by *P. murina* within the extracellular medium. We learned that there were notable differences in the metabolite profiles between organisms that were isolated from the rodent lungs and used immediately afterwards and those that had undergone cryopreservation and subsequently thawed and reconstituted. We gathered that the source of organisms needs to be accounted for in future culture supplementation attempts. The freshly isolated organisms completely depleted glucose available in the media by day 3, whereas cryopreserved fungi had glucose levels comparable to the day of inoculation into the medium. The metabolic byproduct acetate accumulated in greater concentrations and more rapidly in cultures inoculated with the freshly isolated *P. murina* vs the cryopreserved fungi. Such information can be used to direct choices of culture supplements, supplement concentrations, and regimens for re-feeding.

## Discussion

The establishment of a continuous cultivation system for Pneumocystis spp. may lie in our grasp though many parameters must be considered prior to embarking on this ambitious goal. Systematic evaluations that titrate concentrations of appropriate supplements will be necessary. Metabolomics and metabolic flux analyses can be used to guide the amounts and timing of additives. Serious consideration must be given to cellular or other support matrices. Alveolar organoid for studies of pathology and perhaps growth, as well as Air Liquid Interface (ALI) cultures, are exciting avenues that should be explored. Recent genomic, animal model, and transcriptomic studies clearly reveal a requirement for sexual reproduction resulting in the formation of asci for survival of these fungi. Stimulation of the sexual cycle will be paramount in determining a successful culture milieu.

## Author Contributions

MC, NT, SS, and AP contributed to conception and design of the study. NT and SS performed and wrote the ALI and organoid sections. AP conceived and analyzed the metabolomics data. All authors performed the statistical analysis. MC wrote the first and final draft of the manuscript. NT, SS, and AP wrote sections of the manuscript. All authors contributed to manuscript revision, read, and approved the submitted version.

## Conflict of Interest

The authors declare that the research was conducted in the absence of any commercial or financial relationships that could be construed as a potential conflict of interest.
